# The role of companion animals in advanced cancer: an interpretative phenomenological analysis

**DOI:** 10.1186/s12904-022-01050-y

**Published:** 2022-09-16

**Authors:** William R. G. McGhee, Martin Dempster, Lisa Graham-Wisener

**Affiliations:** 1 Belfast Health and Social Care Trust, Belfast, UK; 2 Centre for Improving Health-Related Quality of Life School of Psychology, Queen’s University Belfast, Belfast, UK

**Keywords:** Cancer, Companion animals, End-of-life care, Pets, Psychology, Qualitative research, Palliative care

## Abstract

**Background:**

There is evidence that a companion animal (CA) or ‘pet’ can be helpful during the management of chronic illness. However, the psychological effects of CAs and the mechanism by which they can be beneficial to individuals managing life-limiting conditions is unknown. This study addresses this gap and provides the first examination of the lived experience of CAs among community-dwelling adults with advanced cancer.

**Methods:**

Semi-structured qualitative interview study consisting of a homogenous sample of 6 individuals with an advanced cancer diagnosis, who either self-selected to the study or were recruited through a regional charity that supports palliative and end-of-life care patients in maintaining a connection with their CA. Data were transcribed verbatim and analysed using Interpretative Phenomenological Analysis.

**Results:**

Four superordinate themes occurred in the data: a protective relationship, positive behavioural change, facilitating meaningful social connections and increased loss-orientated cognitions. The findings suggest that CAs offer de-arousing and socially protective supports that mitigate physical and psychological sequalae experienced by people with advanced cancer. However, as their illness progresses, individuals may also experience thoughts related to not meeting their CA’s needs currently and in the future.

**Conclusions:**

CAs provide emotional, practical, and social supports to individuals diagnosed with advanced cancer that can improve individual psychological wellbeing. Consequently, it is important that CAs are considered in advance care planning processes and that services are available to mitigate any negative effects of CA ownership, in order to maximise the benefits CAs confer to individuals managing advanced cancer.

**Supplementary Information:**

The online version contains supplementary material available at 10.1186/s12904-022-01050-y.

## Background

The term Companion Animal (CA) refers to any animal, either domesticated or wild-bred, that is living in a community and is kept for the purposes of company, enjoyment, work, or psychological support [1]. In the United Kingdom it is estimated that approximately 45% of households own a CA, with the majority being either cats or dogs [2]. The potential effect that a CA can have on an individual’s psychological and physical wellbeing is an increasing area of interest among health and social science professionals [3, 4].

A number of studies have advocated for the benefits associated with CA ownership, which include reduced psychological distress, increased social supports, and reduced feelings of isolation and loneliness [3–5]. Predominantly, two causal hypotheses have been proposed to explain this phenomenon. The de-arousing hypothesis suggests that contact with a CA reduces an individual’s physiological arousal and that bottom-up processes subsequently generate positive psychological effects [6]. The stress-buffering social support hypothesis suggests that CAs provide psychological supports that have protective effects against pathogenic consequences [7].

A meta-analysis focusing on long-term health conditions, suggested that CAs provide stress-buffering supports to individuals managing long-term illnesses through mediating social contact and providing direct emotional supports that engender improved mental health and wellbeing [8]. This review concluded that CAs offer communication, emotional, practical, existential, and social supports to individuals, [8]. Accordingly, researchers and clinicians are placing increased emphasis on the importance of CAs within the support system of people with long-term physical health conditions [9, 10]. For example, a mixed-method survey of cancer patients concluded that the majority found their CA had helped them during their illness and recommended that healthcare providers consider patient need relating to their CA [11]. A more recent qualitative study with cancer patients suggested that CAs promote wellbeing by providing companionship, purpose, and emotional support to individuals [12]. CAs may also have negative impacts on those diagnosed with chronic illness [8], which is of equal priority to understand alongside any benefits.

The same understanding does not exist of the benefits and mechanisms through which companion animals may provide support to individuals with advanced cancer and other life-limiting diagnoses [5]. Individuals with advanced cancer can experience heightened complexity in relation to psychological distress [13] and social support need [14, 15], and therefore may benefit from consideration of how a CA may be integrated into care planning to facilitate improved psychological wellbeing. Research indicates that although individuals receiving palliative care cite ‘animals and nature’ as a meaningful aspect of their life [16], they are likely to be unsatisfied with this aspect of their life. Furthermore, in a recent UK policy report [17], 12.7% of the general public cited ‘being surrounded by my personal things/pet’ as being a top three priority in their last days of life, and 14.73% cited the same for their last year of life. A small number of studies of limited quality suggest CAs can confer positive supports to individuals in oncology and palliative care settings [18], although this research has focused on animal-assisted interventions [e.g., [19–23] rather than CAs that are already a part of the individual’s life in the community. Given the extent of CA ownership [2] and the emergent evidence of the benefits of CAs in the wider literature on long-term health conditions [8], there is value in addressing the marked dearth of research examining how CAs are experienced for individuals towards the end of life.

The present study provides the first examination, as far as the authors are aware, of the lived experience of CAs among community-dwelling adults with advanced cancer. The study utilises Interpretative Phenomenological Analysis (IPA) to examine the relationship between CAs and individuals with advanced cancer, in order to generate an understanding that may inform decisions regarding the integration of CAs into advance care planning.

## Methods

### Design

A semi-structured qualitative interview study utilising Interpretative Phenomenological Analysis [IPA; 24] to explore the lived experience of people who have advanced cancer and a CA, and the impact a CA has on an individual while managing their cancer diagnosis. IPA allows for the idiographic examination of an individual’s experience, which can then be critically analysed for implicit meaning [24]. The IPA method promotes the individual’s constructed understanding of the world, where the shared components of individual experience can then be used to infer the wider meaning of said experience [25].

### Identification and recruitment of participants

Homogenous advanced cancer service users were purposively identified based on an analysis of their demographic variables and approached by a UK regional (Northern Ireland) charity, which specialises in supporting individuals with advanced illnesses to retain a relationship with their CA. Additional participants self-selected by responding to digital advertisements placed on social media accounts, with demographic variables assessed to ensure homogeneity of the IPA sample.

Individuals were considered eligible to participate if they met the following inclusion criteria: formal advanced cancer diagnosis, adults (aged ≥ 18 years), capacity to provide informed consent, and ability to read, understand and speak English. Any persons who met the following exclusion criteria, were regarded as ineligible for recruitment into the study: unable to undertake an interview, or their CA is suffering poor welfare.

Eligible people were provided with a written information sheet about the study, where they were informed of the aim of the research. Those who provided verbal consent were contacted over telephone by the main interviewer (WM). The telephone contact provided an opportunity to gain a degree of familiarisation with the interviewer, discuss the project in more detail and, if they consented, schedule a mutually convenient time to conduct an interview.

### Semi-structured interviews

A literature review was undertaken to identify themes for the development of the semi-structured interview [See Supplementary Material 1], as suggested by phenomenological researchers [24, 26]. The semi-structured interview consisted primarily of open questions that generate a descriptive account of experience, with follow up questions included to encourage the participant to provide a more analytic account of their experience.

The interview schedule was developed by the research team (WM, LGW, MD) and consisted of open-ended questions that examined the supports offered by a CA, based on the work of Brooks et al. [8]. The questions aimed to explore the emotional, practical, existential, and negative effects of owning a CA. A single interview was conducted with each participant by a male Trainee Clinical Psychologist (WM) who had no prior knowledge of the participants. Interviews lasted between 60–90 min and were completed either face-to-face or digitally (via Microsoft Teams or Zoom), with the participant at home and their CA present.

### Ethics

All experimental protocols were approved by institutional review board and/or ethical licensing committee of Queen’s University Belfast Faculty of Engineering and Physical Sciences Research Ethics Committee. The study was conducted in accordance with the Declaration of Helsinki and participants completed an informed consent statement prior to completion of the survey.

### Analysis

All interviews were audio recorded and transcribed verbatim by the interviewer (WM). Following transcription, a preliminary analysis and synthesis was completed for each data set. The interviewer also kept a reflective journal, which included field notes recorded during and after interviews. The reflective journal promoted continued reflexivity on the part of the researcher, by allowing them to consider their own experiences and biases during the analysis, in a manner that encouraged methodological rigour [25, 26].

The analysis was guided by Smith and colleagues’ [26] heuristic framework: a serial six-step model for IPA analysis that allows the researcher to move in and out of detail in an iterative manner across interview recordings, transcripts, and field observations. Super ordinate and subordinate themes were generated by the interviewer (WM), which were subsequently ratified by the research team (LGW, MD) to ensure fidelity to the IPA analysis and truthfulness of interpretation.

All participants expressed a positive view toward being involved in any necessary follow-up processes. However, the quality of data was deemed sufficient, and no follow-up was required.

### Reflexivity

Consideration of fore-structures of experience and horizons of significance are integral aspects of phenomenological research [24]. The researcher (WM) approached this study with some personal experience of advanced cancer and selected clinical experiences in psycho-oncology. Likewise, the researcher (WM) had some experience of CAs both growing up and within their current life. Based upon these prior experiences and a familiarity with research literature, a positive preconception was held in regard to the psychological effects CAs may provide their owners experiencing advanced cancer. This was bracketed by the researcher through a curiosity in probing any comments relating to negative experiences of CAs.

### Validity and rigour

In order to ensure that the IPA methodology is transparent, the study is reported within the CORE-Q guidelines [27]. Additionally, the study meets a number of criteria to be considered a rigorous IPA study [28]: Firstly, the study is phenomenological as it focused on the lived experience of CAs on those living in the community with a diagnosis of advanced cancer. Secondly, the study is hermeneutic with the interview schedule developed from the current research base, with relevant psychological theory incorporated throughout. Finally, the study is idiographic in nature, with the themes ratified by the research team consisting of the trainee’s two supervisors (LGW, MD) and a minimum of three participants’ extracts included in each theme.

## Results

A total of 8 charity service users were assessed as meeting the eligibility criteria between August 2019 and March 2021. Overall, 6 service users gave consent to be contacted, with 4 individuals consenting to be interviewed and included in the study. An additional 2 participants self-selected to the study by responding to digital advertisements circulated via social media. A total of 6 participants were included in the study, 3 males and 3 females.See Table 1 for socio-demographic characteristics. The study sample size was concordant with IPA theoretical guidelines and previously published studies, which recommend 4–10 participants [24, 29–31].

**Table 1:** Sociodemographic characteristics of study sample

	**Pet Pseudo-nym**	**Owner Age (years)**	**Pet Type & Age (years)**	**Approx. length of time with Pets (years)**	**Diagnosis**	**Time since Diagnosis (years)**	**Living situation**
Claire	Daisy	47	Dog (3)	3	Breast cancer	8	Living with dependent
Neil	Teddy	66	Dog (2)	30	Prostate cancer	6	Living alone
Agnes	Precious	91	Dog (10)	60	Bladder cancer	13	Living alone
Mac	Buster	43	Dog (4)	40	Anaplastic glioma	7	Living alone
Karen	Rufus & Lilly	58	Dogs (12, 6)	50	Breast Cancer	1	Living with partner
George	Winston	55	Dog (5)	5	Oral cancer	5	Living with partner

Throughout the IPA analysis, 7 subordinate themes emerged concomitant to 4 superordinate themes of protective partnership, positive behavioural change, facilitating meaningful social connections, and loss-orientated cognitions. To ensure transparency in the idiographic nature of the analysis, a coding tree of superordinate and subordinate themes is included below (see Table 2). Excerpts of participant data are included in the text to evidence that the interpretation is grounded in the data, which allows the reader to re-examine the interpretation of the data and assess for internal coherence.

**Table 2:** Coding tree of superordinate and subordinate themes

Superordinate Theme	Subordinate Theme	Quotation	Interpretation
Protective relationship	Providing unconditional positive attachments	‘*She is very affectionate, and she is always showing it … She seems to sense if you’re not feeling well and she seems to stick by you. So she is just like a wee comfort kind of thing’*. (Participant Claire & CA Daisy)	Claire explains that Daisy provides unconditional positive regard and displays emotional congruence towards her. These behaviours foster a human-like attachment that provides a sense of comfort for Claire and increases her overall sense of wellbeing
	Promoting post-traumatic growth	*‘I have a wee bit more time … I can appreciate then the benefits of being out with her. So rather than it being uh an extra job to do, it is maybe a more pleasurable part of my day to be taking her out for a walk’. (Participant Claire & CA Daisy)*	Claire reflects on how Daisy has allowed her to gain a greater appreciation for life. There is a recognition of how Daisy facilitates a greater appreciation of everyday things and encourages Claire to focus on the present moment
	Expressing the emotional effects of illness	‘*I don’t have a deep conversation like when I was with the psychologist or neurologist … it is just how you feel or how I am feeling. … if I am feeling very low or down, I like the dog beside me. I will say come over here Buster and give us a hug’. (Participant Mac & CA Buster)*	Buster offers an outlet for Mac to communicate his feelings while living with advanced cancer. Although Mac highlights that this support is unique to the human-animal bond, he explains that it provides an important support to him that helps him manage his advanced cancer diagnosis
Positive behavioural change	Promoting behavioural activation	*‘Once he gets that walk, oh my mind is content… He helps me in that way because I walk up and down the garden with him’. (Participant Agnes & CA Precious)*	Precious increases Agnes behavioural activation, which improves her wellbeing by meeting Precious’s needs, while also providing Agnes with the benefits of physical activity
	Generating routine and structure	***‘****The main thing is something else to look after and to get out and walk. And you can’t just sit on the sofa and do nothing, because she is needing, she is needing things done and that helps me mentally’. (Participant Claire & CA Daisy)*	Daisy provides Claire with a novel structure and routine. This increased purpose improves her wellbeing and reduces the negative psychological consequences of living with advanced cancer
Facilitating meaningful social connections	Access to practical support Additional social support	*‘The fact that I have dog walkers coming to have a wee talk to. The fact that you yourself are here, you know it breaks up my day for me. … It is nice to get company and company is coming from having him’. (Participant Neil & CA Teddy)*	Teddy generates a variety of connections to others, which Neil explains improves his wellbeing by reducing his feelings of loneliness and isolation by providing outlets that mitigate the negative consequences of the illness
Loss-orientated cognitions	Increased reflection on separation anxiety	‘*So I am glad he is here rather than not being here, but I just worry in case, what happens to him if I go first?’. (Participant Neil & CA Teddy)*	Neil’s reflection on the potential future of their CA, projects his worries about losing Teddy, while also highlighting his own death anxiety
	Focussing on novel limitations	‘*If I seen him not getting walked, I would have a go at myself. I would rather (walk him) than see him closed in all the time, I would go out and it is a wee bit dangerous for me because I stagger’. (Participant Agnes & CA Precious*)	Agnes worries about not being able to meet Precious’s needs. This worry seems interrelated with her attachment to Precious and leads her to engage in increased risk behaviours, driven by a desire to meet the Precious’s needs

### Protective relationship

All participants described a deep and meaningful relationship with their CA, where the primary benefit of the CA is connected to the human-animal bond and the emotional support this affords. In the context of adjustment to advanced illness, this human-animal bond appears to transcend to a greater significance comparable to a human-like attachment [9]. Uniquely, this change is considered a surprising aspect of their illness journey, regardless of whether CAs have been a longstanding feature or are a relatively new part of the individual’s family system.

The significance of the protective partnership is highlighted by the anthropomorphic language utilised to describe the CA and the general status conferred to the CA within the family system of people with advanced cancer. Individuals living with advanced cancer describe their illness as generating barriers that restrict their access to usual emotional supports, which seemingly precipitates the CA’s new significance. The protective relationship superordinate theme contains three subordinate themes: providing unconditional positive attachments, promoting post-traumatic growth, and expressing the emotional consequences of illness.

### Providing unconditional positive attachments

It has been suggested that the beneficial effects of CAs are underpinned by Attachment Theory, [32, 33] and indeed, the protective relationship appears heavily centred on the attachment between the individual with advanced cancer and their animal. Mac, a single man living alone, describes the significance of his CA since being diagnosed with brain cancer:

“Nothing as loving as her. If I am sad, she knows I’m sad and she is very perceptive to me being down or whatever, … I can sense that from her and that gives me a boost and that helps me” (Participant Mac & CA Buster).

Mac explains here that the protective partnership is underpinned by a significant degree of emotional congruence between himself and Buster. Mac suggests that his CA is uniquely attuned to him and that allows them to demonstrate empathy. It is as if Buster fulfils Mac’s need to be understood, which subsequently confers to him emotional support and forms the basis of their protective relationship. The beneficial effects of this positive attachment are heavily centred on Rogerian core conditions of congruence (‘authentic self’, where inner feelings are in line with actual behaviour) and empathy, [34].

The unconditional nature of the attachment between the individual with advanced cancer and their CA is noted as a crucial aspect of the protective relationship. As Claire, a 47-year-old woman with breast cancer, discussing the comfort she finds in her CA explains:

“She just is always so loving and so positive, that if you’re not feeling great and you spend a wee bit of time with her, she just makes you feel a wee bit better *Interviewee becomes emotional*” (Participant Claire & CA Daisy).

Claire explicitly describes the unconditional nature of her attachment with Daisy as an integral aspect of their protective relationship. This significance is emphasised by her description of proximity maintenance, whereby she suggests there are immediate improvements in psychological benefits from simply being close to her CA. It is as if the attachment with the CA instils an increased sense of self-worth in Claire and increases her resilience to the negative consequences of her illness by embodying the Rogerian core condition of unconditional positive regard [34].

Interviewees also described that their unconditional secure attachment to their CA provides them with an increased sense of security and safety while they manage the challenges related to their advanced cancer. As described in Neil’s account below:

“With nobody in the house, he’s there to greet me when I come home and when I go out … He barks to let me know when somebody is coming up the driveway, so he is a wee bit of an early warning system” (Participant Neil & CA Teddy).

Neil’s explanation suggests that his illness generates an increased sense of vulnerability and feelings of loneliness. Neil indicates that his illness has created an interpersonal isolation that the unconditional positive attachment with Buster mitigates against by creating a safe haven and sense of security.

Throughout these accounts the CA is described in a manner that suggests the individual with advanced cancer maintains an almost human-like attachment. The four key features of secure attachment secure base, safe haven, proximity maintenance and separation distress [33] are universally experienced between individuals with advanced cancer and their CA. The unconditional positive attachment between the CA and their owner is an integral part of their protective relationship. This attachment ultimately provides emotional support, which promotes general wellbeing and protects the individual from the negative psychological consequences of their advanced illness.

### Promoting post-traumatic growth

Positive psychological change occurring within an adverse experience is referred to as post-traumatic growth (PTG) and has five facets: relating to others, new possibilities, personal strength, spirituality, and appreciation of life [35]. All interviewees described how their CA has engendered positive psychological change throughout their experience of advanced cancer

“There are lots of frightening things and unpleasant things in our lives at the minute, but if you see her running about, you can’t help smiling… I suppose it is trying to appreciate the things you do have, instead of thinking about the challenges that you have in your life or what is ahead of you … It just brings you back to the moment” (Participant Claire & CA Daisy).

Claire acknowledges how her advanced cancer diagnosis has generated significant psychological distress associated with the unknown aspects of her illness and the general sense of disempowerment this has created. Claire’s account suggests that Daisy provides her with a greater appreciation for the positive aspects in her life, which facilitates her being in the present moment. There is a shift from a loss orientated to an acceptance focused stance, which is indicative of her CA facilitating PTG through a greater appreciation of life and new possibilities [35]. Moreover, the CA seemingly exerts a grounding effect on Claire that encourages psychological adjustment and promotes mindfulness coping behaviours [36].

“He is always here; he is my constant. … He knows when I am down, sad, or happy … He is one of the good things in my life and the fact that I have a good family and a very loving family” (Participant Mac & CA Buster).

Likewise, Mac, while describing the significance of his protective relationship with Buster, highlights PTG in two distinct ways. It is as if his protective relationship has encouraged him to consider his own values and in doing so has engendered a greater appreciation for the different aspects of his life. Secondly, PTG is seen in the description of close relationships, where the CA facilitates an appreciation of Mac’s intimate relationships and an acceptance of his increased emotional vulnerability.

Several interviewees denoted PTG in a divergent fashion, highlighting potential spiritual development associated with their experience of their CA throughout their illness. Increased spirituality has been demonstrated as a significant predictor of psychological adjustment to cancer, regardless of the perceived threat to life [37]. As Karen, a middle-aged woman with breast cancer describes following a period of intensive chemotherapy treatment:

“My relationship with my dogs is much deeper now following being ill and the fact that I realise that spiritually they are very connected to me” (Participant Karen & CA Rufus & Lilly).

Karen specifically states that her CA has facilitated a general spiritual development associated with the depth of their protective relationship. Agnes, an elderly lady diagnosed with breast and bladder cancers, provides a similar narrative indicating a spiritual growth associated with her CA. She explains that she feels a previous CA ‘sent’ her current CA to protect her “*because she has the same antics as the other dog”.*

Unlike Karen, Agnes’s account of spiritual PTG reflects more of an engagement in an existential inquiry relating to the presence of a higher power. The CA in this regard connects the self with a higher presence, which appears to provide Agnes with a sense of wellbeing and emotional support as her illness progresses.

### Expressing the emotional effects of illness

All interviewees explained that a significant aspect of the protective relationship with their CA exists in the ability to express the emotional consequences of their advanced cancer with impunity. As Neil, a 66-year-old man who lives alone and describes himself as increasingly lonely following his prostate cancer diagnosis, describes below:

“On the odd day you get a wee bit down and you start thinking about these things and you wonder what you done wrong … I just talk away, whether he’s listening or not, I just talk away. It’s maybe because I can’t get an answer back from him, but it gets it out of my system for a while” (Participant Neil & CA Teddy).

Neil’s narrative suggests that during the process of trying to understand and accept his illness he experiences low mood and increased feeling of self-blame. He implies that Teddy is an unfettered outlet for him to express the emotional consequences of advanced cancer, while also stressing that the lack of reciprocal communication allows him to engage in a safe process of introspection.

George, a married family man, offers a similar account while reflecting on his illness’s impact on his family. He acknowledges the safety of being able to openly express his emotions to his CA in a way that he cannot do with his family: *“you don’t need to conceal the pain … He has no cares, no worries”*. Although the human-animal bond parallels aspects of human–human relationships [38], it is clear the human-CA protective relationship differs from a human–human relationship, with the latter incurring more considerations that might limit open discourse. In this regard, the CA seemingly offers a unique support to the individual with advanced cancer.

The ability to express the emotional consequences of advanced cancer is a key component of the protective relationship between individual and CA, seemingly embedded in the unconditional nature of the attachment. It provides a direct emotional support to individuals with advanced cancer, allowing patients that safety to communicating the consequences of their illness and express their basic emotional needs. This offers a unique emotional support that provides a stress buffer against the psychological consequences of the advanced illness [8].

### Positive behavioural change

As their illness progresses, all individuals described becoming increasingly susceptible to the negative consequences of their advanced cancer. Their accounts indicate that this manifests as a reduced engagement in meaningful activity, due either directly to physical limitations or as an indirect consequence of mood disturbance.

All participants suggested that their CA precipitated some degree of positive behavioural change during their illness. Their accounts highlighted that the CA creates a sense of purpose in the individual that motivates them to engage in positive behavioural change, which subsequently increases their self-worth and facilitates improved wellbeing.

Individuals with advanced cancer described the CA promoting a variety of positive behavioural change, which can be separated into two distinct subordinate themes: promoting behavioural activation and generating routine and structure.

### Promoting behavioural activation

All interviewees described experiencing psychological benefits from the behavioural activation that occurs primarily as a result of caring for their CA. Below Claire discusses how Daisy has resulted in her re-engaging in exercise, that otherwise she had felt too unwell to participate in:

“One of the things I get the most out of it, is having to take her out walking, being it is keeping me active … Even if you don’t feel like going out you have to take her out. So that is a sort of a practical thing that I have got from it” (Participant Claire & CA Daisy).

Claire states that the behavioural activation promoted by Daisy is one of the most important benefits of a CA while managing an advanced illness. She highlights that the practical support offered by the CA comes through the activity generated in meeting the CA’s need, which also provides an extrinsic motivation to engage in activity. There is a strong inference that this behavioural activation, which would otherwise be neglected, leads to improvements in Claire’s wellbeing and protects her against depressive consequences of her advanced illness.

The promotion in behavioural activation is interrelated with the protective partnership. As George discusses how his advanced cancer has restricted his usual activity, he explains how the protective relationship with Winston provides him with the safety to engage in novel physical activity: “*I got confident to walk around the block… even go out by myself… before I would not go out, so he was getting me out”.* As with Claire, the CA facilitates George’s engagement in positive behavioural change that has a positive effect on his overall subjective wellbeing.

### Generating routine and structure

Participants described how a significant part of the positive behavioural change attributed to their experience of CA during their advanced illness, relates to the routine and structure created by the CA.

In the extract below, Mac highlights the integral role Buster has had in establishing positive behavioural change through generating new routine and structure for him:

“I would worry about me own mental health … if I didn’t have Buster I wouldn’t get out of my bed for a couple of days and that’s never good for anyone. … If I didn’t have Buster some mornings there would be no reason to be getting up … I don’t know where I would be without them” (Participant Mac & CA Buster).

Similarly, Neil described that Teddy has generated positive behavioural change by providing regular routine and structure to their lives:

“Before I was just lounging about the house … Having to make me own tea, sometimes I didn’t feel like making it… But he changed the routine. I have to feed him so I may as well feed myself” (Participant Neil & CA Teddy).

In both of these accounts features of depression are evident. Mac and Neil emphasise the significance of the routine and structure provided by their CA. Both demonstrate awareness into the negative consequences of their absence of routine and increased levels of apathy, yet they describe a lack of motivation to break this negative behavioural cycle. Through providing the routine and structure necessary to care for their CA, the person with advanced cancer is more likely to engage in their own self-care. The CA inadvertently elicits positive behavioural change that the individual would not have had the intrinsic motivation for otherwise. The significance of the CA generating routine and structure is greater for those with an increased sense of interpersonal isolation and appears less prominent in the accounts of individuals living with partners.

### Facilitating meaningful social connections

All individuals expressed the view that their advanced cancer diagnosis generated a sense of increased isolation, which fosters further feelings of loneliness and reduces the perceived availability of social supports. This isolation is a unique aspect of advanced illness that occurs independent of social circumstances; expressed equally by individuals living alone and those living with family or dependents.

Participants highlighted that their CA facilitated their reintegration with others, generating an increased sense of belonging within the individual, which provides them with access to practical and social supports that mitigate the feelings of separation generated by their diagnosis.

Individuals with advanced cancer described their CAs attracting external supports into their lives, which they would have otherwise been without and that they feel to some degree mitigates against the negative psychological consequences of their advanced cancer diagnosis. This would be consistent with literature suggesting that CAs help individuals secure access to practical and social supports [8].

“I’ve got to know a lot more people… It just probably makes you feel a bit more integrated into the whole thing and there probably are people you could ask for support if you needed to” (Participant Claire & CA Daisy).

As a single mother living with a dependent, Claire explains that Daisy facilitates meaningful social connections with others. These connections provide her with the sense of belonging to a community, where otherwise her illness may have led her to feel overly isolated. Claire suggests that these connections could lead to increased social support, which might offer protective factors that mitigate against the negative effects related to the progressive nature of advanced cancer.

Similarly, Agnes suggests that a key benefit of the social connections generated by her CA can be in the emotional support it provides, which can be as simple as others taking an interest in her wellbeing: *“they talk to me, ask me how I am doing”*. While George highlights that the social connections generated can be just as important for those with more established family units because it provides an opportunity to communicate openly about his cancer and worries, “*I talk to people up on the hill… You can talk to a stranger completely”*.

### Loss-orientated cognitions

Individuals with cancer relate their psychological distress to the unknown and a sense of being disempowered by their illness [39]. Alongside the positive roles CAs maintain for those managing advanced cancer, all interviewees indicated that their CA can also result in a significant increase in their experience of loss orientated cognitions. These loss orientated cognitions relate to the CA being an object to project the own worries and could be separated into two subordinate themes: increased reflection on separation anxiety and focussing on novel limitations.

### Increased reflection on separation anxiety

The CA maintains a special place within the individual’s personal system, which relates to the supportive effects of the CA delineated in the superordinate themes: protective partnership, positive behavioural change and facilitating meaningful social connections. Throughout all accounts a concern existed for interviewees as to what might happen to their CAs as their cancer progresses.

“I would have to give her away and that would put me in a worse position because it would genuinely break my heart” (Participant Mac & CA Buster)

Mac describes his fear that as his illness progresses this will lead to an inevitable separation from Buster. It is as if Mac’s account not only highlights his concerns relating to potentially losing Buster and the positive benefits conferred by their relationship. But also, that his interaction increases his focus on his own mortality and increases his own death anxiety.

Likewise, George explains below that during a prolonged period in hospital a facilitated visit from his CA did generate positive effects, but following being separated from Winston he experienced an increased focus on the finality of his advanced cancer diagnosis:

“All of a sudden he was there. Then my wife had to take him away and I suppose that realisation came then, that one day it is going to be for real. That he is not going to see me again” (Participant George & CA Winston).

The fear of loss highlights the significance of their protective relationship, and the benefits Winston confers on George, while also emphasising his projectivity to need support throughout the entirety of the illness. Yet the separation can illustrate the progressive nature of the illness, which increases George’s experience of loss orientated cognitions and furthers his own death anxieties.

### Focussing on novel limitations

The other loss orientated cognitions described by interviewees related to being unable meet the CA’s care needs, attributed to the physical and psychological consequences of advanced cancer. The participants’ accounts suggested an aspect of caring for a CA while managing an advanced cancer diagnosis is the potential emphasis it can place on their own novel limitations.

“Frustrating because I can’t take her out as much. … I have to depend on people to help … I did try and take Buster out a couple of times and I actually took seizures” (Participant Mac & CA Buster).

Mac’s account emphasises feelings of guilt as he perceives his illness has gradually restricted his ability to meet Buster’s needs. Mac further explains that he has engaged in increased risk taking to meet Buster’s needs, which we can infer is an attempt to alleviate the negative effects of being confronted by the physical limitations of his progressive illness.

These functions appear connected to the reciprocity of the positive relationship between the individual with advanced cancer and their CA. As the illness progresses, the individual can no longer meet the CA’s needs as before. The CA then becomes a reminder of their own, inevitably increasing physical limitations, which perpetuates further loss-orientated cognitions.

Participants emphasised that the negative psychological consequences of loss-orientated cognitions were lessened by external social supports, attributed primarily to the CA led reintegration with others. As Neil explains below, a pet charity supports him in meeting Teddy’s needs and provide him with a sense of gratitude for the alleviation of his loss-orientated anxieties:

“The (organisation) has that all in hand you know. If something happens or I go into hospital, you know, he is looked after, so I am very grateful for them” (Participant Neil & CA Teddy).

Additionally, any negative effects highlighted by interviewees would often be followed by a rationalisation or vindication of their CA, through the participant citing that the benefits of their CA outweigh any negative aspects.

“It is certainly not a challenge because of the way I feel about Buster… There are more benefits, than hindrances” (Participant Mac & CA Buster).

As Mac explains here: the unconditional protective relationship he maintains with Buster and the positive effects conferred by the significance of this human-animal bond, justify any challenges from living with a CA while managing advanced cancer.

## Discussion

This study utilised IPA to provide the first examination of the lived experience of CAs among individuals living in the community with a life-limiting condition. Evidence suggests that individuals living with advanced cancer experience a range of psychological sequalae, such as loneliness, low mood and worry for the future [13, 14]. Our findings indicate that CAs mitigate against these negative psychological experiences by providing a range of supports to the individual managing advanced cancer, which encompass emotional, practical, and social supports.

The findings suggest CAs mitigate against the negative consequences of advanced cancer by providing a unique protective relationship that confers significant emotional support to the individual with cancer. The principal component of this protective relationship exists in the unconditional positive attachment between CA and the person with advanced cancer. The positive attachment is evaluated in respect of the congruence and empathy displayed between CA and person, providing amelioration of various psychopathologies [40] and may partially explain how CAs mitigate against the negative psychological consequences associated with advanced cancer.

CAs also provide emotional support to advanced cancer patients by promoting PTG. This is seen in the CA engendering a greater appreciation for life and fostering close personal relationships for the people with cancer. In addition, it was suggested that the CA can also promote PTG through spiritual development. These findings would be consistent with studies investigating the types of PTG experienced in cancer more broadly [41] and could provide further explanation for how CAs promote improved wellbeing among people with cancer.

Our findings indicate that CAs encourage positive behavioural change among people with advanced cancer, by facilitating increased behavioural activation, while also generating routine and structure. Encouraging behavioural activation and changes in negative behavioural patterns, are well-defined therapeutic strategies to treat depression [42] and reduce psychopathologies in cancer [43]. This would explain how positive behavioural changes facilitated by the CA may improve low mood and increase psychological wellbeing among people with advanced cancer.

Alongside these emotional and practical supports, CAs increase access to social supports by facilitating meaningful connections with others. This reintegration with social structures, increases patient access to direct and indirect social and practical supports, which have been postulated as a moderator of life stress [44] and are associated with improved psychological adjustment in cancer [45].

In addition to the positive supports conferred by the CA, our findings demonstrated that CAs also modulate loss-orientated cognitions. These manifest as increased reflection on separation anxiety and a unique focus on novel limitations. Interestingly, any individual discussing a negative effect associated with their CA would provide a rationalisation to mitigate against the negative impact of these loss orientated thoughts, highlighting that a cost–benefit analysis would favour having a CA. Furthermore, across different stages of advanced illness, the findings indicate that the negative effects of loss-orientated cognitions are mitigated by access to social supports. These findings highlight the importance of advance care planning and access to services that maintain and prolong the relationship between a CA and the person with advanced cancer.

**Fig. 1:**
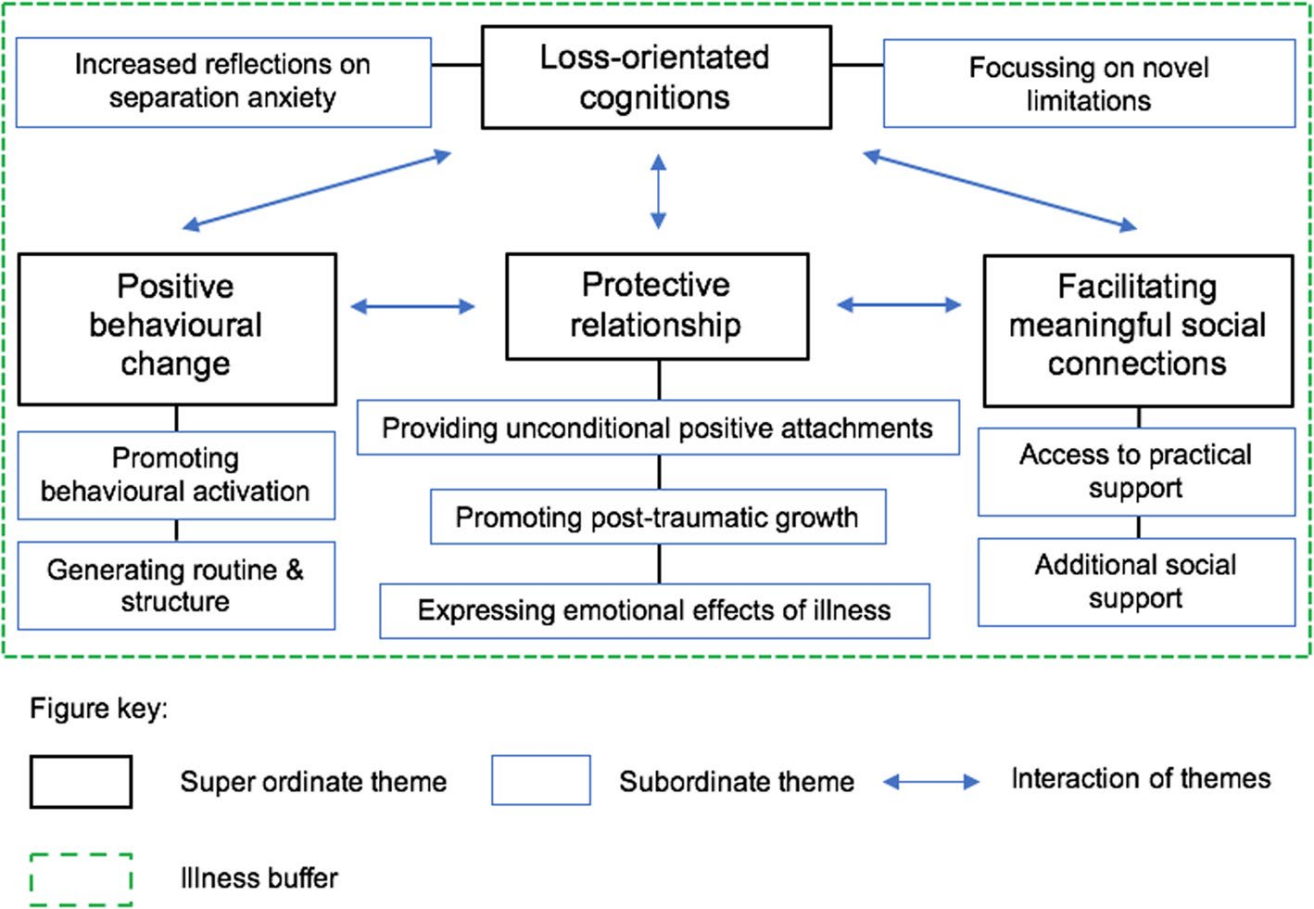
Interaction of superordinate and subordinate themes

The textual accounts suggest that there is likely an interaction between specific superordinate themes (see Fig. 1. for a graphic representation). An example of such interaction can be seen when considering how the protective relationship encourages the individual with advanced cancer to engage in positive behavioural change. The increase in behavioural activation seemingly reinforces the protective relationship between the individual and their CA, which subsequently stimulates further emotional support for the individual and improves subjective wellbeing. Similarly, engaging in positive behavioural change increases the likelihood of the individual’s reintegration with others and the generating of meaningful social connections, which then mitigate against increases in loss-orientated cognitions that exist when owning a CA while living with advanced cancer**.**

Our findings are congruent with previous studies that have evidenced CAs offer primarily positive psychosocial support to individuals with long-term health conditions [8]. The emergent themes exhibit a significant degree of congruence with the wider research, for instance the protective relationship emergent theme encompasses the similar concepts of ‘companionship’, ‘emotional support’, ‘unconditional love and devotion’, and ‘protective care-giver’, noted by Nitkin et al. [12]. Similarly, the discussion of ‘Existential & Spiritual factors’ noted by Nitkin et al. [12] would relate to the subordinate theme of promoting post-traumatic growth, while the emergent theme of positive behavioural change corresponds to the incidents of ‘being in the moment’, ‘purpose & role’ and ‘health and pain management’ suggested in the same works [12]. However, this study did not provide evidence of loss-orientated cognitions, or the potential negative effects associated with CAs that can be experienced by advanced cancer patients. Although this discrepancy could be explained through the different qualitative methodologies utilised across studies, as the enhanced critical incident technique does not allow for a direct comparison to our IPA analysis [46].

Interestingly, whether individuals without advanced cancer may experience similar emergent themes as detailed in this analysis is ambiguous. One might expect that the protective relationship, positive behavioural change and facilitating of meaningful social connections could be a universal human experience of living with a CA. Nevertheless, loss-orientated cognitions, along with promoting post-traumatic growth and expressing the emotional effects of illness, are more likely specific to those individuals experiencing advanced cancer. Albeit it is plausible that these themes and their interactions, as depicted in Fig. 1, could be applicable to other unique transitional life experiences, with similar experiences of CAs noted among individuals diagnosed with posttraumatic stress disorder (PTSD) [31] and borderline personality disorder (BPD) [47].

Wikholm suggested that individuals with PTSD experience their CAs as offering ‘protective partnerships’, ‘beneficial behaviours’, and ‘a role in reclaiming life’ [31]. Comparably Hayden-Evans et al. concluded that people diagnosed with BPD experience their CAs as providing ‘positive emotional attachments’, ‘positive social connections’ and ‘engagement in meaningful activity’ [47]. The protective relationship theme seems analogous to the concept of ‘protective partnerships’ and ‘positive emotional attachments’. Likewise, the theme of positive behavioural change appears equivalent to the idea of CAs offering ‘beneficial behaviours’ and ‘engagement in meaningful activity’, while the facilitating social connections theme matches the ‘positive social connections’ and ‘role in reclaiming life’ themes. Though neither study identified a comparable negative subordinate theme, their contextual narratives suggested that there can be undesirable experiences associated with their CAs and their mental health diagnosis. These similarities across studies highlight the robustness of the IPA analysis and suggest that the findings of the present study have an inherent validity, while supporting the notion that CAs may offer similar positive psychosocial supports to individuals experiencing unique transitional life experiences.

The human-animal bond to an extent appears to mirror a relationship that might be observed between patient-friend or even patient-therapist. A seemingly normal interpersonal dynamic conferring psychological benefits to the patient, can transcend significance when occurring in the context of illness trauma. However, in the absence of adequate additional support structures, this dynamic can lead to an inter-dependency, where separation can be anxiety provoking or even psychologically harmful. Accordingly, the human-animal relationship should be afforded similar consideration to a human–human dynamic, particularly when considering safe-separation and facilitated endings.

In summary, our findings suggest that CAs offer both de-arousing and stress-buffering supports to people with advanced cancer. Initially CAs can have a de-arousing impact on their owners, reducing the immediate physiological impacts that occur while managing an advanced cancer diagnosis. Thereafter, the data suggests that, principally, CAs provide stress-buffering social supports that reduce the negative psychological consequences of advanced cancer and result in improved patient wellbeing. Direct emotional supports are provided through an unconditional protective relationship, while indirect practical and social supports are facilitated through CA led positive behavioural change and reintegration with others respectively. However, while the CA encourages engagement in restoration-orientated processes, it can also generate loss-orientated cognitions that exert a negative effect on people with advanced cancer.

### Strengths and limitations

IPA studies offer a unique method to explore the lived experience of phenomenon with an understanding of the embedded social context of experiences [24]. It is particularly well-suited to examine personal experiences that are often emotional or ambiguous, among small idiosyncratic clinical populations [46]. Accordingly, IPA is a valid method to examine how CAs are experienced by individuals with advanced cancer. However, as IPA studies utilise purposive sampling to generate rich data of individuals’ lived experience of a phenomenon there are debates around the overall generalisability of findings from IPA research and this represents a characteristic limitation of this specific methodology [46]. Smith [48] however emphasises that the validity of a theory can be demonstrated from its applicability within an IPA study, and we have evidenced the relevance of a number of psychological theories and constructs which may be transferrable. Moreover, the sampling method employed may plausibly have generated selection bias, with participants with negative experiences of phenomenon being less inclined to participate in the research and therefore their experiences might not be represented.

Furthermore, although this study did not intend to examine a specific type of CA, the main CAs of participants recruited into the study were dogs and all individuals predominantly discussed their experience of dogs while managing advanced cancer. Albeit a strength in offering a homogenous sample and a more rigorous IPA analysis, this also entails a limitation of the study in that it is not clear how individuals with advanced cancer would experience different types of CA and whether different types of CA may offer individuals distinctive supports during their experience of advanced cancer. We also collected minimal socio-demographic data from the participants, where for the example the homogeneity of the sample in relation to socio-economic status is unclear, a factor which may conceivably heighten negative aspects of CAs for this population.

Nevertheless, our findings converge with earlier studies that have examined the support offered by CAs [8] which would indicate that our results have an inherent validity. This IPA study provides the foundation to inform future high-quality research on the role and impact of CAs across palliative and end of life care populations and settings. For example, quantitative research designs would help to model the predictors and impact of CA ownership on key outcomes for individuals with advanced cancer This is an emergent research area with many uncertainties.

In addition to developing the evidence base on the extent to which CAs impact on psychological wellbeing, subsequent research should address how best to facilitate integration of CAs within advance care planning processes. Firstly, the current study evidences that CAs can be an important part of what an individual considers ‘living well’ towards end-of life, and consideration is needed of how services can best identify and support this need with development of guidance as necessary. For example, a study on advance care planning for individuals with progressive neurological diseases identified provision of a wheelchair to support mobility of an individual who valued being able to walk their dog [49]. Secondly, the current study suggests concern to be present around who will care for the CA when the individual’s health deteriorates or after their death. The Marie Curie booklet on advance care planning makes explicit reference to considering who is able to look after a CA, and signposts to third sector organisations who are able to provide additional support [49]. However, a fuller understanding is needed of the extent to which consideration is currently given to CAs within advance care planning policies and documentation more broadly, and the extent to which bereaved individuals feel their close person’s wishes were able to be provided for.

## Conclusion

This study provides a detailed account of the lived experience of CAs among individuals living with an advanced cancer diagnosis. The unique attachment between CA and the person with advanced cancer provides emotional, practical, and social supports that create an illness buffer against the negative psychological sequelae associated with advanced cancer. Yet a CA can also increase the frequency of loss-orientated cognitions, that have the potential to intensify over the time course of the illness. The findings of this study inform clinical practice by highlighting the importance of considering CAs in advance care planning, as this can maximise the psychological benefits and mitigate any negative effects CAs may pose to an individual throughout their experience of advanced cancer.

## Supplementary Information

**Additional file 1:** 

## Data Availability

The datasets generated and/or analysed during the current study are not publicly available due participant privacy but are available from the corresponding author on reasonable request.

## Abbreviations

CA: Companion animal
IPA: Interpretative phenomenological analysis
CORE-Q: Consolidated criteria for reporting qualitative research
PTG: Post-traumatic growth
