# Supplementary material for: The role of companion animals in advanced cancer: an interpretative phenomenological analysis
Source: BMC Palliat Care. 2022 Sep 16;21:160. doi: 10.1186/s12904-022-01050-y (PMC9479232; doi:10.1186/s12904-022-01050-y)
Supplement: Supplementary file 1 — Additional file 1: [file 12904_2022_1050_MOESM1_ESM.docx]

## **Supplementary Material 1**

**Interview Schedule**

***Exploring the Psychological Impacts of Companion Animals in Self-Management of Advanced Illness***

**Introduction:**

- Introduce self to participant
- Discuss aims of the study
- Ensure that the participant has read and understood the information sheet
- Review informed consent, confidentiality (including limits) and right to withdraw. Check for understanding by asking participant to explain in their own words.
- Agree what the interviewee? should do if they wish to pause or stop the interview.
- Explain under what circumstances the researcher might stop the interview (e.g. signs of distress from the interviewee?).
- Provide the participant with the opportunity to ask any questions
- Begin recording

**Topic guide**

**Types of Support**

What is it like to have a companion animal?

What roles does your CA have in your life?

Has this role changed in any way since your diagnosis?

How does your companion animal help you manage your condition from day to day?

**Emotional Work**

Where would you go to, or who would you go to, when you are worried about your diagnosis?

Does your companion animal help you to cope with your diagnosis in any way?

Is there anything else that you find useful or that helps you when you are worried about your diagnosis or need comfort/support?

**Practical Work**

Living with an advanced condition often means you have to do things more slowly or differently than before, who helps you with this?

How is your companion animal involved in different activities through your day?

**Biographical Work**

What does having a companion animal mean to you at this stage of your life?

How would you describe your relationship with your companion animal?

How has your relationship with your companion animal changed since your diagnosis?

**Communication**

Where would you go to, or who would you go to, to talk to about your diagnosis?

Have you ever talked to your animal about your diagnosis?

**Negative Consequences**

Are there any difficulties with having a companion animal when you have your diagnosis?

***Prompts (can be used under each topic as and when required)***

- Can you tell me a bit more about that?
- How did / does that make you feel?
- What is / was that like for you?
- What did / does that mean for / to you?
- How did / do you make sense of that?
- Describe a specific example / can you think of a specific time when you felt / thought / experienced that?

**Debriefing**

- Ask the interviewee if they would like to add / clarify anything which did not arise during the interview
- Repeat information relating to confidentiality, right to withdraw and storage of information
- Inform the interviewee of how they can receive a summary of the research once it is completed
- Ask the I interviewee about their experience of being interviewed and provide details of support organisations
